# The Effectiveness of a New School-Based Media Literacy Intervention on Adolescents’ Doping Attitudes and Supplements Use

**DOI:** 10.3389/fpsyg.2017.00749

**Published:** 2017-05-09

**Authors:** Fabio Lucidi, Luca Mallia, Fabio Alivernini, Andrea Chirico, Sara Manganelli, Federica Galli, Valeria Biasi, Arnaldo Zelli

**Affiliations:** ^1^Department of Social and Developmental Psychology, Sapienza University of Rome Rome, Italy; ^2^Department of Movement, Human and Health Sciences, University of Rome “Foro Italico” Rome, Italy; ^3^National Institute for the Evaluation of the Education System Rome, Italy; ^4^Department of Education, Roma Tre University Rome, Italy

**Keywords:** Performance and Appearance Substance Use, doping, adolescents, media literacy intervention, attitudes

## Abstract

The aim of the study was to evaluate the effectiveness of a media literacy intervention targeting, for the first time, the specific topic of Performance and Appearance Enhancing Substances (PAESs) use in high-school students. Overall, 389 students (52% male) aged between 13 and 19 years (mean = 16.56 year; *SD* = 1.26) participated to a media literacy intervention (i.e., “*intervention group*”) while 103 students aged between 14 and 19 year (mean = 16.10 year; *SD* = 1.38) were considered as the control group (i.e., “*control group*”). In two separate occasions over the course of six consecutive months, students in both groups filled out a set of questionnaires which included measures of social-cognitive beliefs (i.e., attitudes, subjective norms, intentions) and a self-reported measure of retrospective use of doping (Yes/No) and supplements (Yes/No). Compared to students in the control group (Mean_(time1)_ = 1.96; SD_(time1)_ = 0.85; and Mean_(time2)_ = 2.09; SD_(time2)_ = 0.94), intervention students on average expressed relatively stronger attitudes against doping use over time (Mean_(time1)_ = 2.2; SD_(time1)_ = 0.85; and Mean_(time2)_ = 2.05; SD_(time2)_ = 0.82). Students in the latter group also showed a statistically significant decrease in self-reported supplement use (Use_(time1)_ = 6.7%; Use_(time2)_ = 3.8%; *p* = 0.05, McNemar Test). Interestingly, albeit marginally significant, students in the control group showed a relative increment in the self-reported use of supplements over time (Use_(time1)_ = 4.9%; Use_(time2)_ = 8.7%; *p* = 0.22, McNemar Test). Overall, the media literacy intervention investigated in the present study was effective in decreasing adolescent student’s positive attitudes toward doping use and in reducing the use of legal PAES. These findings supported the generalizability and the usefulness of a media literacy approach in the specific field of PAES.

## Introduction

### The Legal and Illegal PEAS Use and the “Gateway Hypothesis”

Several studies around the world attest that from 0.6 to 5% of adolescents use illegal Performance and Appearance Enhancing Substances (PAES, Mallia et al., 2013). Illegal PAES use is more frequent among male than female adolescents and, to a lesser extent, among older than younger adolescents do (e.g., Yesalis and Bahrke, 2000; Johnston et al., 2007). Furthermore, empirical evidence show that the abuse of performance-enhancing drugs, such as anabolic steroids, is evident across all levels of sports and is likely to implicate people as young as 12 years old (Dunn and White, 2011). The use of illegal PAES is an important health issue, since it poses significant risks for adolescent athletes’ and non-athletes’ health, ranging from risk for reduced fertility to risk for hypertension or psychiatric and behavioral disorders (e.g., Bird et al., 2016). Sometimes, athletes of different levels (elite, amateur, and recreational) and across age groups turn to the use of dietary products (e.g., proteins, amino acids, creatine, multivitamins, and a wide range of herbal products) with presumed ergogenic properties, namely legal PAES, as an alternative to prohibited/illegal substances (Petróczi et al., 2007, 2011). Several studies attested the widespread use of legal PAES across all levels of sport (Maughan et al., 2007; Tscholl et al., 2010), which can reach nearly a 90% level in collegiate sports (Burns et al., 2004) and about 70% in adolescent athletes (Hoffman et al., 2008). In Italy, nearly 7% of 3,400 Italian high school adolescents reported to have used legal PAES in the past 3 months. Even though the legal PAES were perceived by users as a “*safe alternative*” to illegal/prohibited PAES, some scholars (e.g., Metzl et al., 2001) have expressed some concerns about their long-time health consequences in adolescence. Additionally, a growing body of research (e.g., Backhouse et al., 2011; Dodge and Jaccard, 2006) suggested that legal PAES use could represent a “*gateway*” to doping. Research on this hypothesis has shown that self-reported use of legal PAES is associated with higher use of illegal PAES across countries and subgroups (Papadopoulos et al., 2006; Wiefferink et al., 2008; Backhouse et al., 2011; Mallia et al., 2013), as well as over time (Lucidi et al., 2008; Zelli et al., 2010). Along similar lines, a recent meta-analysis clearly showed that legal PAES use had high effect size in predicting doping intentions and actual doping use (Ntoumanis et al., 2014). Even more recently (Barkoukis et al., 2015), some authors addressed the cognitive and behavioral components of the association between legal and Illegal PAES use among adolescent sub-elite athletes. The results of this study supported the “*shared mental representations*” hypothesis, showing that legal PAES use is associated with biased reasoning patterns in favor of Illegal PAES use.

### The Socio-cognitive Mechanisms Regulating PAES Use

In the last two decades, much empirical evidence has clarified the belief systems and social cognitive mechanisms underpinning the intention to use and the actual use of illegal PAES among adolescent athletes. Theoretical frameworks in doping research seem to share the general notion that doping use is a conscious, goal-directed behavior (i.e., performance or appearance enhancement) that involves deliberate reasoning. In line with this notion, doping research had mainly adopted the Theory of Planned Behavior (TPB, Ajzen, 1991) to model the mental processes guiding people’s doping use (Ntoumanis et al., 2014). Broadly speaking, TPB argues that people choose and enact a specific behavior after carefully evaluating the pros and cons of that behavior (i.e., they form a specific behavioral attitude), considering the possible approval or disapproval from significant others in case the behavior is indeed enacted, as well as its perceived prevalence (i.e., Subjective and Descriptive Social Norms, respectively), and reflecting upon the perceived easiness/difficulty to actually enact the behavior [Perceived Behavioral Control (PBC)]. Research utilizing TPB have empirically ascertained the capacity of doping attitudes, perceived behavioral control and subjective norms to predict doping intention and self-reported doping behavior (e.g., Lucidi et al., 2004; Wiefferink et al., 2008; Goulet et al., 2010; Lazuras et al., 2010, 2015), and do so across multiple assessments over time (e.g., Lucidi et al., 2008, 2013; Zelli et al., 2010). These studies have involved a variety of populations, including elite athletes (e.g., Lazuras et al., 2010), gym users (e.g., Wiefferink et al., 2008), and students (Lucidi et al., 2008, 2013; Zelli et al., 2010), suggesting the generalizability of these findings across different samples and settings. Finally, other studies (e.g., Lucidi et al., 2008, 2013; Lazuras et al., 2010, 2015; Zelli et al., 2010; Barkoukis et al., 2013; Mallia et al., 2016) empirically also have shown that TPB effects on doping intentions and behavior do integrate well with other theoretical perspectives [e.g., Social Cognitive Theory (SCT), Bandura, 1991], which highlight other variables, such as self-regulative efficacy (i.e., perceived capacity to cope with or overcome external pressures toward doping) and moral disengagement (i.e., self-serving self-regulatory process that allows people to dope while still believing they are acting morally).

A recent meta-analysis of 63 independent studies (Ntoumanis et al., 2014) examined and confirmed the contribution of TPB and SCT constructs in predicting doping intentions and behavior. Overall, this meta-analysis supported the general conclusion that pro-doping attitudes, biased normative beliefs and prior use of legal PAES are among the most relevant variables regulating the choice of using doping substances. Within this perspective, several scholars (e.g., Barkoukis, 2015) claimed the need to start from and use this empirical evidence in order to develop effective anti-doping interventions.

### Intervention Programs on PAES Use and Their Efficacy

As recently pointed out also by Haw (2016), the few published studies that have examined the effects of anti-doping education programs have reached conflicting and inconclusive results (Backhouse et al., 2014). Overall, any of these studies can be traced back to one of three traditional approaches to anti-doping education. The first is the “scare-based” approach. Studies following this approach have confirmed its inefficiency, and even its possible boomerang effect, like observations in the field of drug use prevention (e.g., Goldberg et al., 1991a,b). Furthermore, a recent review of this literature (Petróczi et al., 2014) pointed out that many interventions focusing on negative health risks or fear appeals have been criticized for exaggerating the risks associated with doping use and for deliberately disregarding the experiences of those doping users who appear or feel healthy. A second approach encompasses specific training programs focusing on ethical decision making. A study (Elbe and Brand, 2016) evaluating the efficacy of this approach yielded unexpected findings, which showed that young athletes’ attitudes toward performance enhancement became more positive after receiving doping ethics-based education.

Finally, most of the educational programs evaluated in literature have used knowledge-based approaches stemming from cognitive research and theories of reasoned action and planned behavior. Overall, also these studies have shown contrasting results (e.g., Backhouse et al., 2007, 2014). The studies have mainly shown that knowledge about drugs, specific banned substances, and alcohol issues are improved after the intervention (Backhouse et al., 2009). However, research on knowledge-, attitude-, and intention-based interventions in some case has failed to provide generalizable results (e.g., Fritz et al., 2005). The most comprehensive anti-doping interventions within this approach were certainly the ATLAS programs (Adolescents Training and Learning to Avoid Steroids; see Goldberg et al., 1996; Goldberg and Elliot, 2005) and the ATHENA programs (Athletes Targeting Healthy Exercise and Nutrition Alternatives; see Goldberg and Elliot, 2005; Elliot et al., 2008). ATLAS and ATHENA are two gender-based interventions that were designed to prevent the use of legal and illegal performance enhancement substances (PESs) and organized to be peer-led and coach-facilitated. Recently, in a meta-analysis evaluating the effectiveness of randomized controlled trials of studies analyzing the two programs, Ntoumanis et al. (2014) showed a very small, albeit statistically significant, reduction in doping intentions but no change in doping behavior. As recently pointed out by Barkoukis et al. (2016), several factors should be considered in order to explain these relatively weak effects. First, both ATLAS and ATHENA adopted a very broad health promotion perspective, addressing a wide range of health behaviors (e.g., training and eating patterns, tobacco and alcohol use) alongside PES use. Furthermore, it should be noticed that ATLAS and ATHENA were conceived and developed about 20 years ago, and their core ideas and contents necessarily could not address or take into consideration the many doping research findings and developments of the last two decades.

The above considerations implicitly call for the need of “upgrading” interventions by focusing on PAES use and by grounding protocols to the research findings on the socio-cognitive mechanisms regulating PAES use. This position is in line with that of European Union’s experts in Doping Prevention in Recreational Sports, who recently have recommended to develop national preventive interventions on doping that can target adolescents and young adults, and who also have highlighted the need of published controlled studies investigating anti-doping interventions (Backhouse et al., 2014). To fill out this gap, recently Barkoukis et al. (2016) investigated the effectiveness of a school-based intervention in promoting anti-doping culture among adolescents, by targeting their perceptions of sport values, social norms and attitudes toward PAES use in sports. The results showed that intervention group participants (*n* = 109) after the intervention reported significantly lower levels of attitudes only toward legal PEAS, and higher norm salience than control group (*n* = 109). However, no significant differences were observed on attitudes toward illegal PAES (doping) between the two groups.

### A Media Literacy Approach to Health Education Programs

A recent meta-analysis (Se-Hoon et al., 2012) has considered and examined the effectiveness of a broad array of media literacy education interventions across several health-related behavioral domains. This contribution shows that media literacy education programs have never been implemented in the behavioral domain of PAES use. Broadly speaking, this literature also shows that, media literacy empowers people to be critical thinkers about and creative producers of an increasingly wide range of media and technology messages using image, language, and sound. As information and communication technologies transform society, they impact our understanding of ourselves, our communities, and our diverse cultures, making media literacy an essential life skill for the 21st century. Media literacy education promoting health among youth is designed to involve them in a critical examination of media messages as an attempt to prevent the internalization of thin ideals and reduce social comparisons with the portrayed models (Levine and Piran, 2004). The objectives of media literacy programs are vast and include, for example, promoting healthier body image and eating behaviors, fostering self-confidence, learning advocacy skills, educating about the prevalence and etiology of eating disorders, and encouraging constructive alternatives to restrictive dieting and drugs as a meaningful way to manage weight. Its efficacy is predicated on the assumption that if youth have sufficient critical thinking skills they will be able to resist the influence of these messages. Several recent studies have tested the ability of media literacy education interventions to teach youth how to analyze media messages and to improve their choices on a variety of health topics. Meta-analytic studies indicate that media literacy interventions have positive effects (*d* = 0.37) on outcomes including media knowledge, criticism, perceived realism, influence, behavioral beliefs, attitudes, self-efficacy, and behavior (Se-Hoon et al., 2012).

### The Present Study

Departing from scholars’ calls (e.g., Backhouse et al., 2014) for studies investigating anti-doping interventions, the aim of the present study was to evaluate a media literacy based intervention targeting, for the first time, the specific topic of PAES use in high-school students.

The choice to carry out the program at school was mainly due to the fact that adolescent athletes and non- athletes represent a high-risk group for both illegal and legal PAES use (e.g., Mallia et al., 2013). Consequently, the school is an optimal setting for accessing a large and representative sample of both types of adolescents. Furthermore, school-based interventions focusing on doping issues may have a potential impact at grassroots level of sport, as they may successfully reach both young athletes at the onset of their career in sports and young people engaging in amateur sports. It is also relevant to create an anti-doping culture also outside of conventional sports settings, since some empirical evidence has shown that the self-reported use of legal and illegal PAES in adolescent non-athletes can be higher than in adolescent recreational and competitive athletes (e.g., Wanjek et al., 2007). The choice of designing and carrying out a media literacy intervention in the domain of PAES use primarily relies on the robust evidence summarized above suggesting that media literacy interventions have the capacity of eliciting significant changes in processes and behaviors implicated in other health behavioral domains (Se-Hoon et al., 2012).

## Materials and Methods

### Schools and Participants

Data is based on 389 students aged between 13 and 19 years (who participated to the school-based intervention (i.e., “intervention group”) and on 103 students aged between 14 and 19 year who were treated as the “control group.” All students which participated to the study were enrolled in one of 30 high schools that were evenly distributed across Italy. The participating students were identified via a convenience sampling procedure. Each school participating to the study contributed with a group of students which ranged from 8 to 17 students. Students’ parents were fully informed in advance about the aims of the study, and written consent was obtained by both the schools and the students’ parents. To all participants, the research and assessment were presented as focusing on “sport practice, lifestyle and beliefs about doping substance use” and self-report questionnaire data were collected during school hours in two separate sessions over the course of about 6 months. In each session, the assessment lasted approximately 10 min. The intervention was carried out for the intervention group between the first and second assessment.

Initially, 430 adolescent students (52% male, age mean = 16.56, *SD* = 1.26) were enrolled in the “intervention schools,” whereas, 114 adolescent students were enrolled in the “control schools” (age mean = 16.10, *SD* = 1.38). All these students provided first wave questionnaire data collected during school hours. Most of the students from the intervention schools also participated to the 12 intervention sessions in the following school months (i.e., the attendance rate across the 12 sessions was of 90.1%). After the intervention sessions, students provided second wave questionnaire data, and 389 and 103 adolescent students provided completed questionnaire data across the two waves for the intervention and control groups, respectively (i.e., over 90% of the students in both conditions provided completed data).

The study was approved by the Ethics Review Board of the Department of Social and Developmental Psychology, “La Sapienza” University of Rome.

### Characteristics of the Intervention Program

The intervention was designed with the core goal of involving youth in re-conceptualizing what realistic objectives might be with respect to sport performance and to the pursuit of personal esthetic goals through sport. In doing so, the present research heavily relies on Potter’s (2004) cognitive approach to media literacy. This approach overall calls the attention on media literacy skills that relies on three interlocking cognitive processes, namely, (1) the acquisition of knowledge structures in diverse areas concerning the media (e.g., media contents, media effects), (2) the boost or reinforcement of diverse cognitive skills which are at the basis of media literacy (e.g., analysis, evaluation, abstracting), and (3) one’s individual capacity to re-frame his or her awareness and goals through the active effort of discounting or minimizing the possible detrimental effects of media.

On these grounds, the present research designed an intervention program that consisted in 12 90-min sessions, which took place in students’ classrooms and that were scheduled twice a month throughout the school year. During the intervention activities, students actively participated to seminars and meetings with different professionals, such as:

(1) communication experts, who mainly provided feedback and arguments for highlighting the role media messages can have in promoting dysfunctional beliefs on (a) sport as the means to unrealistic objectives, (b) advertising which alters and falsifies body images and portrayals and may contribute to esthetic ideals that are unrealistic and impossible to meet, and (c) doping as a necessary means to achieve outstanding performances (two sessions);
(2) pharmacology experts, who discussed and provided students with correct information on the side effects of doping substances and on the ways correct nutrition and lifestyle allow one to reach optimal performance, especially considering the misinformation that often is carried out by media (two sessions);
(3) high level sport athletes, who actively engaged students on issues concerning the moral and ethical implications of doping substance use and on the ways media may disregard or minimize these issues (two sessions);
(4) sport psychologists, who primarily addressed and provided discussion arguments on the ways beliefs and other mental strategies may help students in re-framing his or her awareness and sport related goals, in order to counteract temptations toward doping use (two sessions).

During most of the sessions’ time, students were strongly encouraged to share their personal views with each other and work together in group, and students discussed and addressed issues freely and with little interference on the part of experts. During the last four sessions, students actively and in full autonomy worked on the development of a media message and a sensitization campaign against doping use, and they were instructed to think about their age peers as the target group of this type of campaign. In fact, past evidence (Banerjee and Greene, 2006) suggested that media literacy interventions that involved audience actively (e.g., with discussion or production activities) are more effective than those that involved audience passively (e.g., with lessons only). The activities of the last four sessions were supervised by psychologists and communication experts.

All the experts (communication and pharmacology experts, high-level sport athletes and sport psychologists) which participated to the intervention sessions relied on the same material and stimuli in conducting their session activities. Furthermore, the session protocols that all experts used were initially organized and drafted by the principal investigators of the study. As a result, a training manual was prepared and distributed to all experts in order to standardize as much as possible the intervention activities.

### Assessments

All participating students filled out twice the set of questionnaires described below. For intervention group students, the assessments took place prior to and after the intervention sessions described earlier. For control group students, the assessments took place prior to and after a series of physical education or health education classes that were part of the normal school schedule.

The set of questionnaires included measures that were used in prior studies (e.g., Lucidi et al., 2008, 2013; Zelli et al., 2010) and that included the assessment of social-cognitive beliefs and of a self-reported retrospective (i.e., past 6-month) use of a series of doping substances (Yes/No) and supplements (Yes/No).

Attitudes toward doping use were assessed by asking adolescents to express on a five-point scale to what extent their “use of illegal substances to improve sport performance or physical appearance would be…” useless/useful, foolish/wise, undesirable/desirable, negative/positive, harmful/beneficial, and advantageous/ disadvantageous. Item scores were aggregated into a single score, for which higher values indicated more positive attitudes about doping (Cronbach’s α = 0.64).

Subjective norms were assessed by asking adolescents to indicate their personal experience’s correspondence with the two following items: (1) to what extent significant others would approve their use of illegal substances to improve sport performance or physical appearance, and (2) to what extent they were convinced of meaningful others’ approval. For each of the two items, students responded on a five-point scale ranging from 1 (“not at all”) to 5 (“completely”). Item scores were aggregated into a single score, for which higher values indicated greater normative social pressure to use doping substances (Cronbach’s α = 0.72).

Intentions were assessed though two separate doping intention items measuring the likelihood of using doping substances in the next months (i.e., “How strong is your intention to use illegal substances to improve your sport performance or your physical appearance in the next months?,” and “What is the probability that you will use illegal substances to improve your sport performance or your physical appearance in the next months?”) Responses were recorded on a five-point Likert scale ranging from 1 (“not at all strong/likely”) to 5 (“very strong/likely”). Item scores were aggregated into a scale mean score, for which higher values indicated stronger doping intentions (Cronbach’s α = 0.81).

To measure doping and supplement use, as in prior doping research (Lucidi et al., 2008; Zelli et al., 2010), students were asked to indicate which substance, if any, they used “in the last 3 months with the aim of enhancing their athletic performance or improving their physical appearance”. The list of supplements included creatine, carnitine, and amino acids. The list of illegal products was based on the list adopted by the International Olympic Committee and accepted by the Italian National Olympic Committee, including anabolic- androgenic steroids, peptide hormones (i.e., growth hormone or human chorionic gonadotrophin) and stimulants. For each doping substance and supplement, a 0/1 score was first assigned to each respondent to indicate non-use/use of the substance in the last 3 months.

### Data Analysis

In order to determine whether students in the intervention group reported, as compared to their counterparts in the control group, the expected changes in their beliefs about doping use, questionnaire data were analyzed using a series of 2 (Group: Intervention vs. Control Intervention) × 2 (Time: Pretest vs. Posttest) repeated measures ANOVAs. Possible changes in doping and supplements self-reported use were evaluated through McNemar Test.

## Results

**FIGURE 1:**
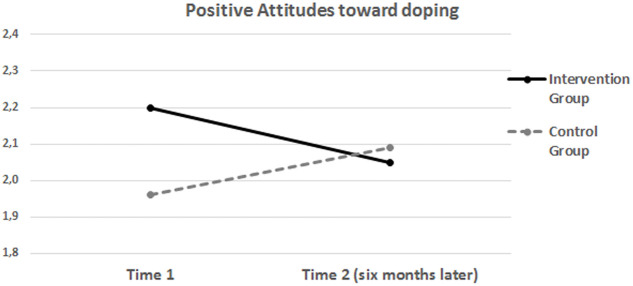
**Positive attitudes toward illegal PAES use (i.e., doping) in intervention and control groups across 6 months**.

One of the key ANOVAs results of the study was a statistically significant Group by Time effect on students’ positive attitudes toward doping (*F*_(1,490)_ = 7.02; *p* = 0.008; $\eta_{p}^{2}$ = 0.02). The ANOVA analyses yielded null main effect for Group (*F*_(1,490)_ = 0.45; *p* = 0.831) and for Time (*F*_(1,490)_ = 1.60; *p* = 0.206). The key Group by Time effect is summarized in **Figure 1**. As one can see from the figure, students in the intervention group showed, over time, a decrease in positive attitudes in favor of doping use (i.e., Mean_(time1)_ = 2.2; SD_(time1)_ = 0.85; and Mean_(time2)_ = 2.05; SD_(time2)_ = 0.82). In contrast, control group students seemed to show an opposite pattern over time, as their attitudes in favor of doping use slightly increased over time (Mean_(time1)_ = 1.96; SD_(time1)_ = 0.85; and Mean_(time2)_ = 2.09; SD_(time2)_ = 0.94).

There was no significant Group x Time effect on subjective norms (*F*_(1,490)_ = 0.001; *p* = 0.973) and on behavioral doping intentions (*F*_(1,490)_ = 0.802; *p* = 0.369). However, it is worth noting that, while prospective behavioral intentions remained virtually the same across assessments among intervention students (Mean_(time1)_ = 1.64; SD_(time1)_ = 0.99 and Mean_(time2)_ = 1.65; SD_(time2)_ = 0.95), control students over time expressed slightly stronger behavioral intentions (Mean_(time1)_ = 1.51; SD_(time1)_ = 0.79 and Mean_(time2)_ = 1.61; SD_(time2)_ = 0.95).

Finally, separate McNemar’s non-parametric analysis of students’ self-reported substance use in the two groups showed, respectively, a statistically significant decrease in supplement use among intervention group students (Use_(time1)_ = 6.7%; Use_(time2)_ = 3.8%; *p* = 0.05), and a statistically non-significant change in self-reported substance use among control group students (Use_(time1)_ = 4.9%; Use_(time2)_ = 8.7%; *p* = 0.22 for McNemar Test).

## Discussion

The literature has clearly ascertained the use of both illegal and legal PAES among adolescents (e.g., Mallia et al., 2013). Furthermore, a large body of evidence has attested that TPB variables (i.e., attitudes, social norms) and the use of legal PAES increase the risk of intending to use and using illegal PAES (e.g., Ntoumanis et al., 2014). Finally, despite findings showing that specific education (i.e., media literacy) interventions may have a positive impact on some TPB variables (e.g., behavioral beliefs, attitudes, and behaviors), there has never been education programs of this sort focusing on the specific context of PAES use (Bergsma and Carney, 2008; Se-Hoon et al., 2012).

In order to overcome this gap, the present study evaluated the efficacy of a media literacy intervention which specifically targeted illegal PAES use (i.e., doping) among high-school students. In line with the literature on other health-related domains (Se-Hoon et al., 2012), authors of the present research developed an intervention program that contemplated 12 90-min sessions, which were conducted during the school year in two sessions per month. The first eight sessions of the program focused on helping adolescents to develop skills for recognizing and critically evaluating the potential damaging effects of sport images in the media, which tend to suggest and support unrealistic views about the body. The program was thus also designed to help students to modify their views about these negative and distorted media images and, indirectly, to elicit positive or alternative ways to conceive or develop media messages concerning sport and doping use. During the sessions, different professionals (e.g., communication experts, pharmacologists, sport psychologists, sport experts) led group activities eliciting a critical analysis of core issues from students, without however, interfering with the ways students would freely organize or work together on specific tasks. Also in line with previous media literacy interventions (Banerjee and Greene, 2006), the last four sessions provided students with the actual opportunity to develop and produce in full autonomy media messages and sensitization campaigns against doping use targeting age peers.

Overall, the findings of the school 6-month intervention suggest that it was effective in eliciting positive attitude changes in students’ views of illegal PAES and doping substances. More importantly, the intervention also seemed to exert an effect on reducing students’ self-reported use of legal PAES. These results have additional value if one considers that, within the same assessment timeframe, control group students reported an increment in their positive attitudes toward doping substances and showed no change in the percentage rate of those who reported using legal PAES.

The present research is, to date, the first evidence of media literacy efficacy in the domain of PAES in adolescence, and it extends prior media literacy literature in supporting the core notion that media literacy may in fact be a valid means in the reduction of risky health behaviors (Austin et al., 2005) and/or in the promotion of stronger attitudes and views against those behaviors (e.g., Gonzales et al., 2004; Banerjee and Greene, 2006, 2007; Austin et al., 2007). It also implicitly argues in favor of the notion that media literacy interventions are capable of fostering healthy outcomes.

The present research also has important implications for the doping literature, as its findings acknowledge that media literacy interventions can be effective in changing two critical factors contributing to doping intentions and the use of doping substances, namely, doping attitudes and the use of legal PAES (e.g., Ntoumanis et al., 2014). The fact that the present research provided evidence of a reduction in self-reported use of legal PAES (i.e., supplements) is of particular importance, insofar several scholars (e.g., Metzl et al., 2001) expressed clear concerns about the possible long-term health consequences of their use, despite clear evidence that adolescents tend to view them as a “safe alternative” to illegal/prohibited PAES (Petróczi et al., 2007, 2011). The present research also has great value for the specific literature on illegal PAES and the “gateway hypothesis,” as both to differing degrees stress the strong ties between legal and illegal PAES (Dodge and Jaccard, 2006; Papadopoulos et al., 2006; Lucidi et al., 2008; Wiefferink et al., 2008; Zelli et al., 2010; Backhouse et al., 2011; Mallia et al., 2013). Finally, the findings of the present research, by showing that an intervention on the use of illegal PAES (doping substance) could indirectly affect and reduce a different behavior, namely, students’ self-reported use of legal supplements, are consistent and support Barkoukis et al. (2015) hypothesis that people who are users of using legal supplements may hold mental representations of illegal substances that are similar to the mental representations of those who use doping or illegal substances.

Despite these significant results, it is to note that the intervention did not influence, at least in a statistically meaningful way, students’ subjective norms or their prospective intentions to use doping substances. The null effect concerning subjective norms is not surprising and is utterly in line with the efficacy research findings of media literacy interventions in other health behavior domains (Se-Hoon et al., 2012). This null finding may also be partly due to the fact that the intervention was not specifically designed to modify students’ perceptions of interpersonal or external pressures toward doping use. However, it is plausible that the intervention may have worked to modify students’ capacity to manage and overcome external pressure toward doping (e.g., doping self-regulative efficacy), a possibility that future studies may carefully consider and address. With respect to students’ prospective doping intentions, the null effect of the intervention is probably due to a two-fold consideration, namely, that the assessment of doping prospective intentions in high school students was premature, given the young age and that, as the variable’s mean score suggest, the statistical effect was estimated in presence of a “floor effect.”

Finally, some concluding remarks on effect sizes. The key result of a Group by Time interaction was associated with an effect size (i.e., $\eta_{p}^{2}$ = 0.02) that would typically be considered relatively “low” in magnitude (see Cohen, 1988). This consideration would legitimately suggest to be cautious in drawing strong conclusions or practical implications. This notwithstanding, it also seems that the effect size of the present finding is in line with other literature and studies, be they specific to the analysis of the effectiveness of media literacy interventions or the effectiveness of interventions in the domain of doping use (see Se-Hoon et al., 2012; Ntoumanis et al., 2014; Barkoukis et al., 2016). Future studies, perhaps addressing longer timeframes between intervention and its possible effects, are needed to clarify the meaning and value of interventions’ effect sizes.

### Limitations and Future Directions

The present investigation has some limitations that need to be addressed. Firstly, the intervention was implemented in school settings, limiting the possibility of generalizing its findings to sport-related contexts, such as juvenile sport teams or various levels of sport involvement. Secondly, students’ assignment to intervention or control conditions was not rigorously randomized, thus raising issues of internal validity. Thirdly, this research was utterly based on self-reported data, and the lack of any objective measure of students’ behavior (e.g., students’ time spent in browsing anti-doping websites) hindered the strength of the intervention efficacy. Additionally, as a recent meta-analysis suggested (Se-Hoon et al., 2012), media literacy interventions tend to have greater effects on media-relevant outcomes, as one would expect (e.g., knowledge and realism). This is plausible, as media-relevant effects are the natural outcomes of media literacy interventions. The present research did not include media-relevant outcomes, a weakness that future studies will need to address.

An additional limitation of the study is concerned with the general consideration that its assessments included a relatively small set of measures, as compared to the sets of measures typically utilized in the studies drawing from TPB (e.g., Lucidi et al., 2008). This limitation is primarily due to the time constraints that the participating schools imposed to the research team. As a result, the choice of which set of measures should be included was guided by several considerations, mostly concerning the predictive value of those variables that have been traditionally considered in the existing TPB doping literature. It also follows that future studies should necessarily include a broader roster of variables that encompass other theoretical frameworks and constructs (e.g., self-regulatory efficacy, moral disengagement).

Finally, there are methodological issues that need to be mentioned. One is related to the possibility that our findings were biased, as the study did not measure or control for effects within or across schools (i.e., the unit of analysis was the adolescent student). The relatively small number of schools involved in the present study did not permit a more rigorous multi-level analysis. The second methodological issue is concerned with the adoption of a convenience sampling procedure and the corresponding caution in addressing or presuming the internal and external validity of the study.

## Conclusion

Notwithstanding these limitations, the present study has meaningful implications for educational agencies involved in promoting doping-free sport and in building an anti-doping culture also outside of conventional sports settings. The present research evaluated a media literacy intervention in the specific domain of PAES use, outside the typical sports settings and amongst non-athlete adolescents. These last features are extremely relevant since – as outlined by the “Fitness against Doping report” (European Health Fitness Association [EHFA], 2012) – recreational sport organizations are at the moment unprepared and lack strategies and initiatives to prevent doping use in the general public, especially among younger exercisers. Thus, efficacious anti-doping interventions in school settings are a viable way to reach a large audience of young people and, hopefully, reinforce their anti-doping beliefs and attitudes.

In sum, we feel that our intervention represents a ready and valid preventive “*tool*” that educational agencies and school institutions seeking to promote doping-free sport may consider and include within their common health promoting activities.

## Author Contributions

All the authors substantially have equally contributed to the development and preparation of the manuscript. Furthermore, all authors have approved the final version of the manuscript. Finally, the authors have agreed to be accountable for all aspects of the manuscript in ensuring that questions related to the accuracy or integrity of any part of it are appropriately investigated and resolved.

## Conflict of Interest Statement

The authors declare that the research was conducted in the absence of any commercial or financial relationships that could be construed as a potential conflict of interest.
